# The Teamwork Model: Proposing a Model for Studying Interprofessional Healthcare Teams

**DOI:** 10.15694/mep.2019.000081.1

**Published:** 2019-04-12

**Authors:** Matthew D'Angelo, Ronald Cervero, Steven Durning, Lara Varpio

**Affiliations:** 1 Uniformed Services University of the Health Sciences

**Keywords:** interprofessional, healthcare, teams

## Abstract

This article was migrated. The article was marked as recommended.

Patient safety is a preeminent healthcare concern in modern medicine. In the years since *To Err is Human*, researchers have found that the number of preventable deaths far exceeds 98,000; more accurately, 400,000 patients die each year from preventable healthcare errors. To combat the evolving patient safety crisis, a variety of organizations from Institute of Medicine to World Health Organization have called for the development of interprofessional healthcare teams. Interprofessional healthcare teams and teamwork have been a topic of discussion for over 40 years. And while some Interprofessional healthcare teams have been shown to be beneficial in some settings the success of these teams is not universal nor achieved in all healthcare settings. In short, research has found that interprofessional healthcare teams both improve and impede patient care. Building on this contradictions of interprofessional healthcare teams and teamwork, we present a model for the conceptualization of teamwork that could be readily applied to clinical experiences. This model is informed by the interprofessional healthcare team literature and relevant theories, and we believe will enable us to examine authentic interprofessional healthcare team interactions and identify moments when team interactions were breaking down, and reasons why those breakdowns were happening.

## Teamwork Failure: A Case Scenario

A 33 year old male patient was admitted to a major medical center for open femur fracture following a ten foot fall off a ladder. Although the patient’s previous medical history was unremarkable, the repair of the fracture was complicated by an acute vascular injury that required resuscitation following severe blood loss. The primary members of the perioperative team included an attending surgeon, a senior surgical resident, an operating room nurse, a nurse anesthetist, an anesthesiologist, and a surgical technician. On this particular day the attending surgeon was running late and the team attempted to expedite the induction of anesthesia to reduce the delay and ensure the room closed on time to reduce staff overages. The patient was “fast tracked” through the preoperative holding area and taken to the room before the attending surgeon arrived. The timeout was performed after induction by the operating room nurse. Unbeknownst to the operating room nurse the patient name and medical record number was incorrect and the name of another patient. This old label had been erroneously placed on the patient chart, an oversight that no other members of the team noticed or cross checked. The patient information on this old label was incorrect information for the patient on the operating table. Not only was the patient’s name incorrect, but so was his blood type. Tragically, the otherwise healthy patient unexpectedly died postoperatively due to an acute hemolytic transfusion reaction from the administration of ABO incompatible blood. According to the post mortem root cause analysis, all members of the interprofessional healthcare team (that included physicians, nurses and technologists) were professionally competent employees of the medical center, had worked together, and had been long standing members of the organization. Despite the individual competence of the team members, and the familiarity of the team to one another, it was determined that failed teamwork played a central and critical role in the patient’s death.

## Introduction

Unfortunately, adverse patient events such as the one described in this scenario happen all too often in healthcare settings. Understandably, then, patient safety has become a preeminent healthcare concern in modern medicine. This heightened awareness is also due, in no small part, to the Institute of Medicine’s (IOM) 1999 landmark report *To Err is Human: Building a Safer Health System.* The *To Err is Human* report highlighted that more than 98,000 patient in the United States died each year as a direct result of preventable healthcare errors. ( Kohn, 2000) While the report elevated the patient safety conversation to a national level and created a “call to arms” by patients, advocates and the healthcare industry, the report has been critiqued as grossly underrepresenting the true scope of this public health epidemic. ( James, 2013) Arguably, *To Err is Human* merely shone a light on the proverbial “tip of the iceberg.” The findings in the report represent merely a quarter of the actual preventable patient deaths at the hands of the U.S. healthcare system. ( James, 2013) In the years since *To Err is Human*, researchers have found that the actual number of preventable deaths is far greater than 98,000; instead, more accurate estimates report that over 400,000 patients die each year from preventable healthcare errors. ( James, 2013) Although organizations and the healthcare industry at large have made significant strides to improve patient safety through organizational changes like procedural time outs ( Hazelton *et al.*, 2015) and automated safety checks aimed to reduce iatrogenic morbidity and mortality, ( Haynes *et al.*, 2009) the healthcare system continues to grapple with patient safety problems.

The public and healthcare industry were recently reminded of this by the IOM’s 2015 report *Improving diagnosis in health care.* In this report the IOM asserts that missed and delayed diagnoses account for delays in treatment and directly impact patient morbidity and mortality. ( Balogh, 2015) Indeed, a recent BMJ article estimates that medical errors is the third most common cause of death in the United States. ( Makary and Daniel, 2016) Unfortunately, despite nearly two decades of awareness, and deliberate actions directed towards improving patient safety, data demonstrate that human factors continue to be a significant cause of preventable healthcare errors and a tragic loss of life. ( Commission, 2015)

To combat the evolving patient safety crisis, a variety of organizations from Institute of Medicine (IOM) to World Health Organization (WHO) have called for the development of interprofessional healthcare teams (IHT). ( Kohn, 2000; *Framework for Action on Interprofessional Education & Collaborative Practice*, 2010; Gilbert, Yan and Hoffman, 2010; Balogh, 2015) IHT and teamwork have been studied for over four decades, both in the US ( ’Core competencies for interprofessional collaborative practice: Report of an expert panel.,’ 2011) and internationally. ( *Framework for Action on Interprofessional Education & Collaborative Practice*, 2010) And while IHTs have been shown to improve patient care ( Firth-Cozens, 2001; M. A. West and Lyubovnikova, 2013), improve patient safety ( *Framework for Action on Interprofessional Education & Collaborative Practice*, 2010), reduce cost ( Ross, 2000), reduced visits and hospitalization rates (Sommers *et al.*, 2000), lower staff absenteeism and turnover ( Rosenstein, 2002), and prove to be a more effectively use resources and improve patient satisfaction ( MA. West, Dawson, J.F., Admasachew, L. and Topakas, A., 2011), these benefits are not universal and are not achieved among all IHTs and in all healthcare settings. (M. A. West and Lyubovnikova, 2013) In fact, IHT failures from non-technological sources like decision-making, cooperation, problem solving and team member miscommunication have been reported as major barriers to the effectiveness of IHTs. ( Vincent, 2005; Mishra *et al.*, 2008; Bleakley, 2013) Indeed, research into physicians’ and patients’ views of errors reports that the failure of health professionals to work together or communicate as a team is the third most important cause of preventable medical errors. ( Blendon *et al.*, 2002)

The effectiveness, or utility, of IHTs is a subject of ongoing and intense study for healthcare organizations, scholars and clinicians. With more than 15 definitions of a “team” described in the context of healthcare, attempts to understand, model, and develop IHTs is a challenge. ( Bleakley, 2013) This complexity is compounded further when we take into consideration that the criteria for determining the evaluation of team “effectiveness” are highly context dependent. In other words, the best-practices for in one setting are not necessarily transferable to others (e.g., advantages reported from research conducted in surgical settings may not be transferable to out-patient settings; the value added from interventions in the UK may not be realized in the US ( ‘Core competencies for interprofessional collaborative practice: Report of an expert panel.,’ 2011).

Due to the ambiguity surrounding the definition of the IHT and the lack of specificity on how to define an “effective” IHT, scholars regularly return to foundational questions about the causes that contributed to the medical error. For instance, in considering the scenario described at the beginning of this manuscript, we might ask: How is it that an incorrect blood product was dispensed from the blood bank by a trained technician, traveled to the operating room by a trained staff member, and was checked and verified for correctness by two team members (as is the process in this clinical context)? How could this blood product traverse multiple layers of safety checks to be deemed “safe” and administer to a vulnerable patient? Are the professionals in this scenario truly a “team” or are they in actually a loosely affiliated group of individuals connected by geography and a shared patient? How can a group of competent individual care providers fail to achieve collective competence as a team? ( Lingard, 2012)

In this manuscript, we synthesize the literature on IHTs that addresses these foundational questions and we propose a model for conceptualizing teamwork. Specifically, relying on a recent literature, we (i) review the foundational definition of the word “team”, (ii) examine the essential characteristics of successful IHT, (iii) describe a theoretical model that operationalizes the description of a team and characteristics of successful IHT, (iv) describe the theoretical underpinnings of the model, and (v) describe how the model can be operationalized to analyze and understand team performances.

## Definition of a Team

What is a team? *Teams* and *teamwork* are vaguely described in the literature and encompass a wide variety of meanings. Etymologically, the word *team* arises from Germanic languages and refers to a group of animals yoked together to collectively pull a carriage, move equipment or soil, etc. ( Tse and Ho, 2014) The yoke served as a harness that was constructed from wood and rested upon an animal’s shoulders enabling multiple animals to pull or work together cooperatively towards a desired goal. From this description, a “team” can be defined as two or more individuals who work cooperatively through a framework to successfully complete a task. The components of the “basic team,” then, are (1) multiple individuals, (2) who work interdependently, (3) through a framework that supports collaboration, towards the (4) achievement of a shared goal. While these four components may appear straightforward, they are the foundation for successful teams and, when not aligned, is often a reason for unsuccessful, and failed team collaboration.

## Essential characteristics of a team

Mapping the characteristics of effective IHTs is an important but elusive goal for researchers and academics who study teams. In reviewing the healthcare literature, it is clear that many authors have attempted to describe the essential characteristics of a successful IHT. And while this effort has increased the understanding of IHTs, definitive accounts of the characteristics of successful teams remains tenuous. ( Nancarrow *et al.*, 2013) Scores of authors have identified a range of characteristics for IHTs. While characteristics differ across contexts studied, researchers commonly acknowledge a core set of qualitatively similar characteristics of effective IHTs. These characteristics are: common goals, effective communication, and respect among team members. ( Campion, Papper and Medsker, 1996; S. Mickan and Rodger, 2000; S. M. Mickan and Rodger, 2005; *Framework for Action on Interprofessional Education & Collaborative Practice*, 2010; D. P. Baker *et al.*, 2010)

Over the last two decades, research suggests that teamwork can be defined by interrelated knowledge, skills, and attitudes (KSAs). ( Cannon-Bowers, 1995; E. Salas, Bowers, C.A., Cannon-Bowers, J.A., 1995; D.P Baker, Gustafson, S., Beaubien, J.M., Salas, E, Barach, P., 2003; D. P. Baker, Day and Salas, 2006) Through extensive work within the healthcare domain ( Howard *et al.*, 1992; Gaba *et al.*, 1998; Flin and Maran, 2004; Healey, Undre and Vincent, 2004; Thomas, 2004; D. P. Baker, Day and Salas, 2006) researchers have identified eight competencies that are present in successful IHTs. These eight competencies incorporate the three characteristics of successful teams and expands upon them. Further, teams that have these KSAs have been shown to outperform teams that did not have the KSA’s. ( Smith-Jentsch, 1996; Leonard, 2001; E. Salas, Burke, S.C., Bowers, C.A., Wilson, K.A., 2001; O’Shea, 2003; D. P. Baker, Day and Salas, 2006) Salas, et al. (2004) define these KSAs as: (1) Leadership ( Cannon-Bowers, 1995; D. P. Baker, Day and Salas, 2006; Eduardo Salas, Sims and Burke, 2016), (2) Backup Behavior ( Porter *et al.*, 2003), (3) Mutual Performance Monitoring ( McIntyre, 1995), (4) Communication ( McIntyre, 1995), (5) Adaptability ( Cannon-Bowers, 1995; D. P. Baker, Day and Salas, 2006), (6) Shared Mental Models ( Jonker, van Riemsdijk and Vermeulen, 2011), (7) Mutual Trust ( D. P. Baker, Day and Salas, 2006), and (8) Team Orientation. ( E. Salas, Sims, D.E., Klein, C., 2004; D. P. Baker, Day and Salas, 2006) Table 1 presents a summary of these essential characteristics.

**Table 1.** Essential Characteristics of Successful Teams

KSA	Description	Relevant Citations
Leadership	Ability to direct, motivate, plan, assign, and coordinate team activities. Establishes team climate.	D. P. Baker et al., 2006; Cannon-Bowers, 1995; E. Salas, Sims, D.E., Burke, S., 2005
Backup Behavior	Ability to anticipate other team member’s needs	Porter et al., 2003
Mutual Performance Monitoring	Know other team members role, provide feedback, redistribute work.	D. P. Baker et al., 2006; McIntyre, 1995
Communication	Effective Information exchange	McIntyre, 1995
Adaptability	Ability to adjust strategies and performance to evolving situations	Cannon-Bowers, 1995
Shared Mental Models	Understanding team organization, shared goals.	Jonker, 2011
Mutual Trust	Members will perform their responsibilities to the team, and protect team members.	D. P. Baker et al., 2006
Team Orientation	Take others behaviors and solutions into account. Put team above self.	D. P. Baker et al., 2006

## The Teamwork Model

The eight KSAs identified above provide important insights into the characteristics of a successful IHT. One could argue, however, that this list is incomplete. While these eight KSA’s describe important team characteristics, they do not consider the individual team members ability to perform effectively. These team-level characteristics assumes that individual team members are capable of functioning within the team. These KSAs fail to account for the clinical competence, emotional wellness, and physical ability of the individual team member. ( Welder, 2016) This is a significant omission since there is substantial focus in the literature investigating the individual’s competence to perform a skills or demonstrate knowledge. ( Bleakley, 2006; ten Cate, Snell and Carraccio, 2010; Lingard, 2012) In this literature, a team member’s professional competence is viewed, at least in part, as an important contributing factor to his/her ability to perform as a team member. (ten Cate, Snell and Carraccio, 2010) In other words, a key knowledge, skill and attitude consideration for IHT performance must ask: Does the team member have the requisite skills to perform with the team? Proxy measures like professional licensure can, and often does, serve as indicators of individual competence or ability. Unfortunately, while such proxy measures may demonstrate professional clinical competency, an individual team member’s emotional wellness and or physical ability is often overlooked as a contributor to team performance. A clinically competent, yet emotionally distraught or physically injured team member could be a liability and reduce the efficacy of an IHT.

Therefore, we propose that the eight-part KSA model for successful IHT should be augmented with an additional KSA. A ninth KSA, individual competency, should be added to the list of successful team characteristics and should account for (1) the clinical skill that the individual brings to the team, (2) the emotional state that the individual brings to the setting and (3) the individual’s physical ability that is brought to a task.

**Figure 1.**
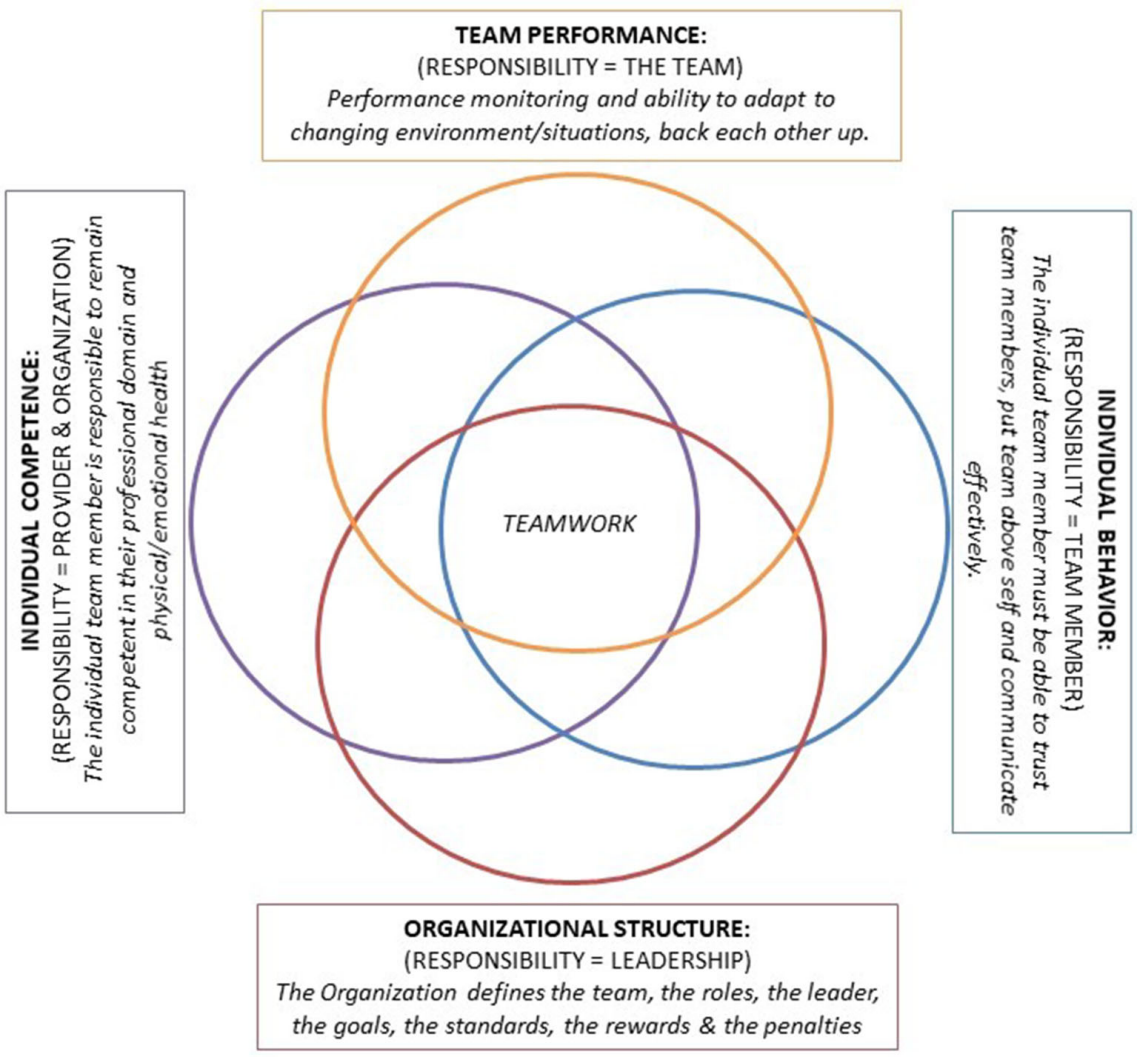
Illustration of the *Teamwork Model*

Based on these nine KSAs, we have developed the *Teamwork Model.* The *Teamwork Model* organizes the previously identified essential team characteristics (KSA’s) and integrated individual competencies. We propose that successful teamwork is a result of four interdependent domains that contain the nine KSA’s (see Figure 1 for an illustration of the *Teamwork Model*). The interdependent domains are: (1) Organizational Structure, (2) Individual Competence, (3) Team Performance Skills, and (4) Individual Interactions. The *Teamwork Model* can be visualized as four Venn diagram circles. Each circle represents an individual domain, which each domain including a sub-set of the nine KSA’s associated with successful IHT performance. Table 2 lists the organization of the *Teamwork Model* and subdivision of the KSA’s within each domain. The four interdependent domains of the *Teamwork Model* are as follows:

• **The Organizational Structure Domain** is purposefully located at the base of the diagram. We propose that a successful team must be grounded with a clear charter (or defined purpose), roles, leadership, goals, standards, rewards and penalties. The larger organization within which the team is housed (e.g., hospital, government, health professions accrediting body) is responsible to define, appoint membership/roles, and empower the members of the team so they may be positioned to be successful.
• **The Individual Competence Domain** encompasses the responsibilities of the individual team member and includes the individual’s clinical, emotional, and physical competence to serve on the team. It is the individual’s responsibility to maintain this competence. While Individual Competence is a duty of the member, the Organization has the responsibility to regulate individual team membership and is entrusted with the authority to measure or ensure fitness for those who serve on the team.
• **The Team Performance Domain** encompasses the team-level considerations including the team’s collective ability to adapt to changing environments, monitor team performance, and provide backup to team members when they fail to meet expectations. The team and the individual team members are collectively responsible for this domain. These KSA’s are acquired through socialization and practice with one another.
• **The Individual Interaction Domain** relates to an individual’s interaction with the team. The team member must learn to trust other members, be able to communicate with other members of the team, and develop a collective orientation to the team where the goals of the team outweigh the goals of the individual.

**Table 2.** KSA distribution between Teamwork Model domains

Individual Competence	Organizational Structure	Individual Team Performance Skills	Individual Behavior
**Clinical Competence to Perform**	Leadership	Performance Monitoring	Mutual Trust
**Emotional Competence**	Shared Mental Model	Adaptability	Team Orientation
**Physical Competence**		Backup Behavior	Communication

## Theoretical Underpinnings of the Teamwork Model

The *Teamwork Model* relies on and is congruent with two theoretical frameworks that have been previously used to investigate IHTs. They are: Lingard’s conception of Collective Competence and Engestrom’s Cultural Historical Activity Theory.

### Collective Competence

The concept of collective competence “reflects growing attention in the social and organizational spheres to healthcare’s natures as a complex system.” ( Lingard, 2009) It broadens the concept of competence that has traditionally held an individualist orientation, to include a collective participation orientation. Thus, in terms of IHT, collective competence highlights how healthcare teams are deeply interconnected, so much so that “a change or weakness in one part of the system affects both other parts and the performance of the whole.” ( Lingard, 2009)

### Activity Theory

The *Teamwork Model* is also conceptually grounded inthe philosophical perspective of Cultural-Historical Activity Theory (CHAT) since it considers IHT’s KSA through the complex goal directed social encounters of a team. CHAT was conceptualized by the Finnish educational researcher Yrjo Engestrom and is based on the foundation of Lev Vygotsky and Aleksei Leont’ev Activity Theory ( Engestrom, 2000; Yamagata-Lynch, 2010; Witte and Haas, 2016). According to Activity Theory (AT), humans interact with the environment through the use of “tools”. Activity theorists’ argue that individuals and groups use tools to influence reality.

**Figure 2.**
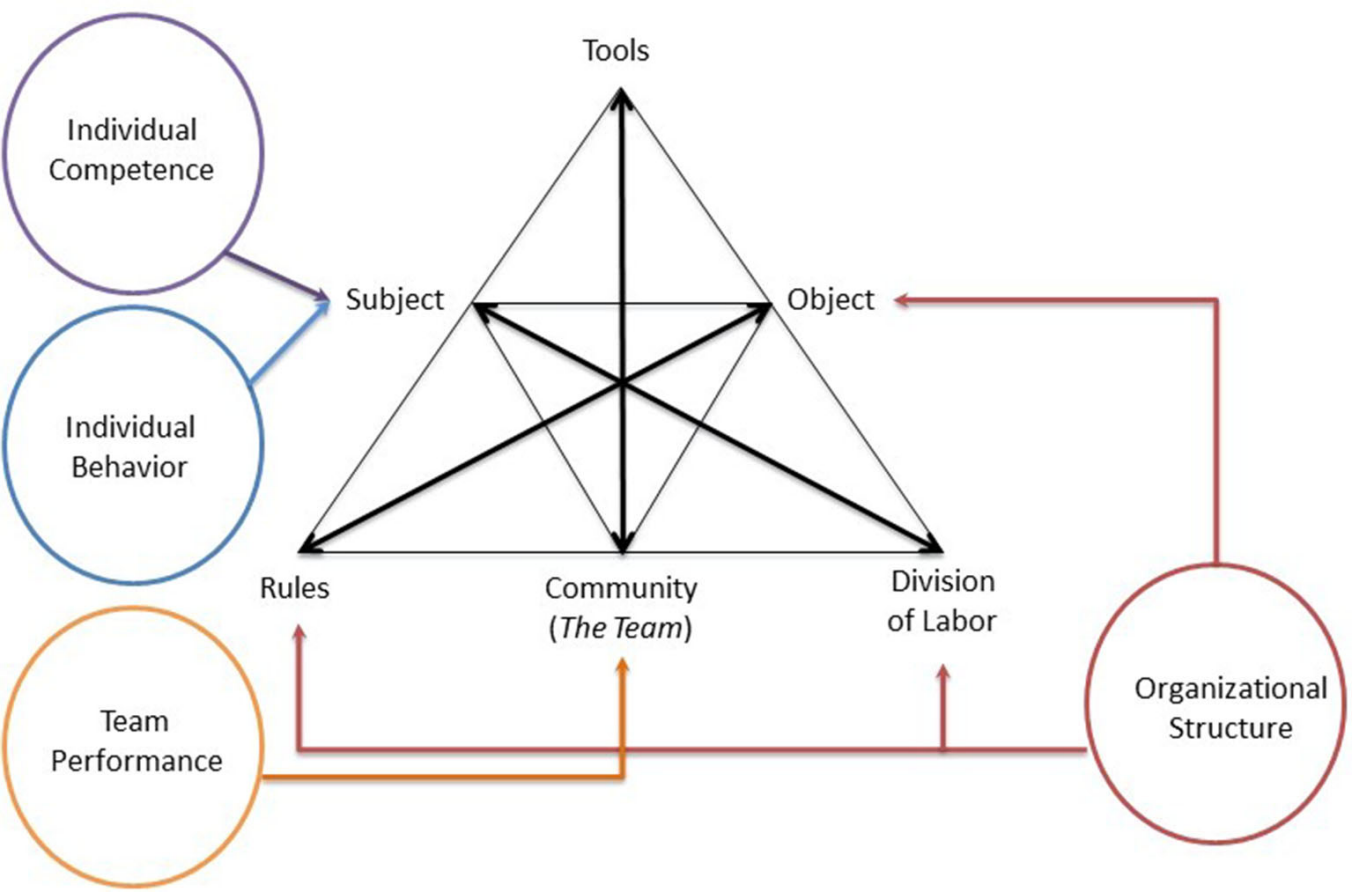
Engestrom’s Activty System and the Teamwork Model

**Figure 3.**
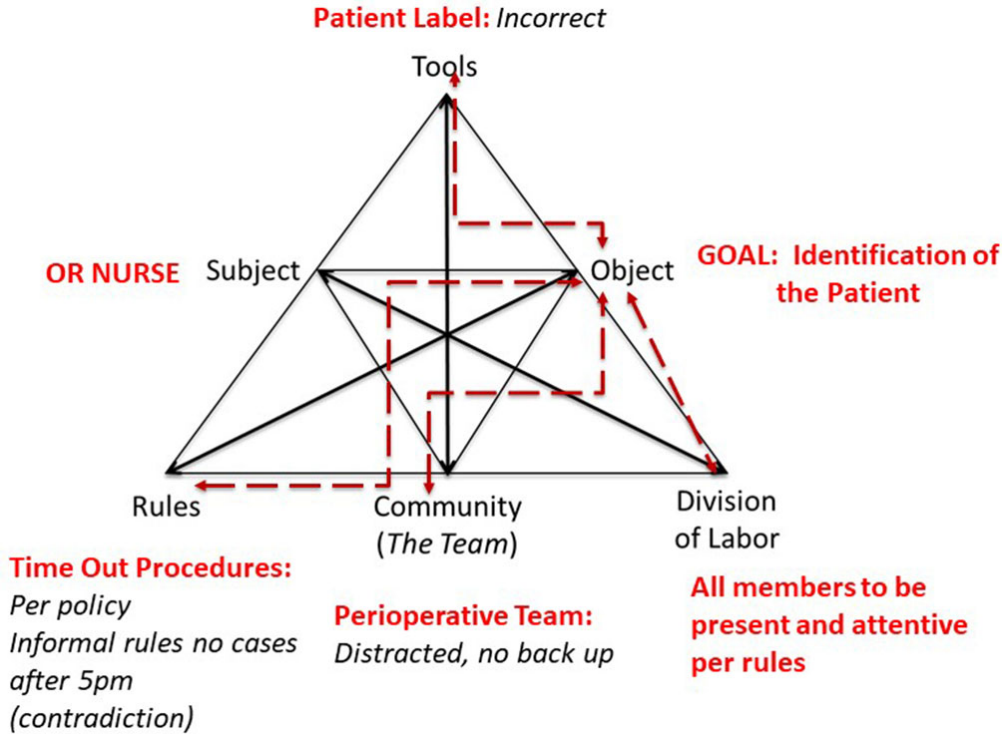
Alignment between Engestrom’s CHAT and teamwork

Engestrom’s CHAT builds upon the theoretical foundations of AT and provides a succinct visualization of the interactions between the team member (i.e., subject) and the complex environment in which they will be required to work. Figure 2 represents Engestrom’s model of the activity system as incorporating the four domains of the *Teamwork Model.* As Figure 3 illustrates, the *Teamwork Model’s* four interdependent domains are aligned with Engestrom’s CHAT. The CHAT “subject” is the *Teamwork Model’s* individual team member, including the Individual Competency and Individual Interaction domains. The “community” element of Engestrom’s model represents the team itself and aligns with the *Teamwork Model’s* Performance Behaviors domain. The *Teamwork Model’s* Organizational Structure domain encompasses AT “rules”, “division of labor” and “objects”.

## Using the Teamwork Model to analyze team performances

We propose that the *Teamwork Model* can be used to support the analysis of team performance to identify strengths and weaknesses. It is important to note that IHTs can function effectively without all domains being fully incorporated and without all KSAs being fully realized; however, teams that work outside the domains and KSAs described in the *Teamwork Model* may suffer from “near misses” in patient safety, delays in function or other characteristics of a poorly functioning team.

The *Teamwork Model* presumes that interdependent domains of KSA’s are required of an effective IHT. Through the *Teamwork Model,* we propose that when the domains of KSAs are coordinated and work interdependently, teams will function more successfully and effectively. Furthermore, we acknowledge thatit is important to consider teams in the context of their work domains. While we propose that successful IHTswill share similarities across a wide range of contexts, the component elements of the *Teamwork Model* will likely need to be weighted differently in different contexts.

**Figure 4.**
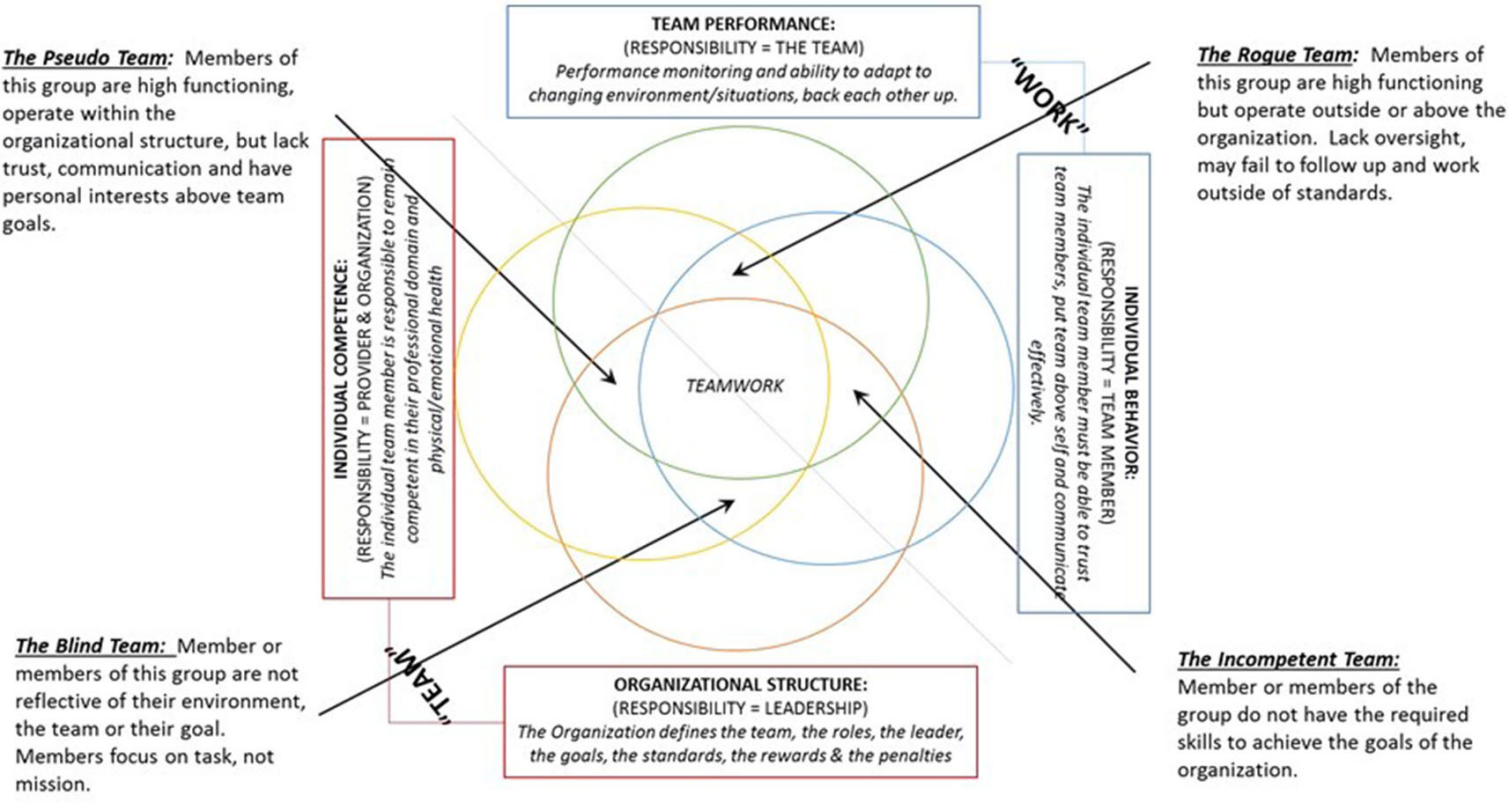
Problematic team dynamics

We suggest that the *Teamwork Model* provides a means for assessing team characteristics and team performances. Using the *Teamwork Model,* we identify four (4) kinds of problematic team dynamics that may be produced when one of the model’s interdependent domains is not present. Figure 4 illustrates where these four problematic team dynamics fall in the *Teamwork Model.*

***The Blind Team*** *i*s a group of affiliated individuals that lacks characteristics (KSA’s) from the Team Performance Domain. Specifically, a Blind Team is one that is unable or poorly adapts to changes in their environment. This group does not monitor its performance or provide backup behaviors. While members of this group are individually competent, demonstrate good individual interactions, and follow the organizational mandates, they focus their attention to individual tasks and are not an integrated team.

***The Pseudo Team*** is a group of affiliated individuals that lacks characteristics (KSA’s) from the Individual Interaction Domain. This group may be high functioning, operate within the organizational structure, but it lacks trust, inter-team member communication, and a collective orientation to the objective. Members of this group may be unable to delegate, work well with each other, or may put individual goals or motives above other members or the goals.

***The Rogue Team*** is a group of affiliated individuals that lacks characteristics (KSA’s) from the Organizational Structure Domain. Like the Pseudo Team, members of this group may be high functioning but operate outside or above the organization. This group lacks oversight, may fail to follow up, and can work outside of the standards and rules.

***The Incompetent Team*** is a group of affiliated individuals that lacks characteristics (KSA’s) from the Individual Competence Domain. These groups likely struggle to function and to meet requirements due to inadequate individual skills, emotional or physical requirements required to achieve the goal or objectives.

## Applying the Teamwork Model to the Case Scenario

If we apply the *Teamwork Model* to the case scenario at the introduction of this manuscript, we can describe the team’s problematic performance as an instance of a Blind Team. On this day, team members were competently performing their jobs and focused on their own domain or sub goal of the team. No member of the team recognized that the operating room nurse was reading the name of a patient from an old patient label in a hospital chart. Due to this oversight, the unconscious and vulnerable patient on the table was “transformed” into another person whose blood type of record was different than that of the patient on the table. The team failed to recognize the error because they were distracted from the time out while performing individual skills. Due to informal rules, the team attempted to expedite the procedure to avoid have a late running room. As a result, the patient’s admitting documents were not fully scrubbed leading the OR nurse to read an incorrect patient identifier. In other words, the team performed as a Blind team because members of this group were not reflective of their environment, the team or their goal. Members focused on individual task. This blind OR team failed to provide performance monitoring and backup behaviors. The team members assumed the OR nurse would correctly identify the patient. From this assumption the preoperative nurse was allowed to read an incorrect patient name without challenge. This team was blinded by assumption to this mistake. Through the simple oversight of an incorrect name a highly functioning group of individuals set the course for a disastrous event subsequently killing an otherwise healthy young man. As a result, the goal of correctly identifying the patient, along with all his relevant information including blood type, was not achieved and the patient was incorrectly identified for surgery.

Team failures such as the one described in this scenario are not unfamiliar to healthcare providers. Healthcare is complex, requiring professionals to provide care while balancing the needs of their patient with professional, organizational and personal influences. By all accounts, the individual members of this IHT performed competently as individuals. The patient was intubated, positioned and surgery initiated. The team followed organizational policies; performing the time out and checking blood before it was administered to the patient. Individual interactions were successful. But as a team, they were unable to be successful. Based on the *Teamwork Model* we can identify the team’s functioning as a Blind Team and so can better understand how the team failed to perform as a successful collaborative IHT. With these insights, remediation can be appropriately directed ensuring that this error will not affect another patient in the future.

## Conclusion

The *Teamwork Model* we present synthesizes into one model the major characteristics required for successful teamwork and adds to these characteristics an individual competence characteristic. The *Teamwork Model* incorporates individual-focused and collective-focused competencies, and builds on CHAT’s attention to the individual, social, and material contexts that inform IHT performance. Organized as a Venn diagram, the *Teamwork Model* emphasizes integration and interdependence of the competency domains, highlighting that the “ideal” or successful team represents all characteristics. Grounded in the theories of Collective Competence and CHAT, the *Teamwork Model* is organized into four domains and provides a lens to functionally assess IHT performance. The *Teamwork Model* allows for organizations, teams, and individuals to analyze team performances and identify problematic team behaviors. Through this model, we believe that IHT performance can be evaluated and in some cases remediated to improve team function, team success and patient care.

IHT represents a paradigm shift in modern healthcare delivery and has been recognized as an important means for reducing iatrogenic sequelae and improving patient outcomes. But we cannot expect that simply introducing IHTs in healthcare can mitigate patient injury and improvements to care. Indeed, successful IHTs are composed of collectively competent individuals who work together to complete a shared goal. We hope that the *Teamwork Model* can provide a framework for putting into action the important discoveries already made about IHT towards the goal of developing interprofessional healthcare team that successfully function together to minimize cost, and improve the care of patients in the modern healthcare system.

## Take Home Messages

The term team is commonly used in healthcare practice, yet the quality and effect of teams varies widely. This manuscript proposes a model that incorporates 4 domains and 9 competencies for successful teamwork. Through this model provides the foundation to evauate teamwork performance.

## Notes On Contributors

The authors are faculty members at the Uniformed Services University of the Health Sciences. The views expressed are those of the authors and do not reflect the official policy or position of the Uniformed Services University of the Health Sciences, the Department of the Defense, or the United States government.

Matthew D’Angelo is an Associate Professor of Nursing and and a graduate student in the Health Professions Education Program at the Uniformed Services University.

Ronald Cervero is a Professor and Associate Director for Remote Campus Education for the Graduate Programs in Health Professions Education.

Steven Durning is a Professor of Medicine and Pathology and Director, Graduate Programs in Health Professions Education (HPE).

Lara Varpio is a Professor and Associate Director of Research, Graduate Programs in Health Professions Education.
