# Supplementary material for: The Teamwork Model: Proposing a Model for Studying Interprofessional Healthcare Teams
Source: MedEdPublish (2016). 2019 Apr 12;8:81. [Version 1] doi: 10.15694/mep.2019.000081.1 (PMC10712620; doi:10.15694/mep.2019.000081.1)
Supplement: Peer review history

# Reviewer report: Reviewer response for version 1

**DOI:** 10.21956/mep.19807.r29810

**Published:** 2019-11-18

**Authors:** Sateesh Babu Arja

**Affiliations:** 1 Avalon University School of Medicine

This review has been migrated. The reviewer awarded 3 stars out of 5 A very well written paper. The authors detailed the conceptual and theoretical framework for a team work model for studying inter-professional healthcare teams. The literature review is up to date and relevant. This manuscript could be useful for the academic administrators who are working on developing inter-professional and trans-professional educational curricula for undergraduate and post-graduate medical education.

Reviewer Expertise:

NA

No decision status is available

# Reviewer report: Reviewer response for version 1

**DOI:** 10.21956/mep.19807.r29809

**Published:** 2019-05-04

**Authors:** Hossam Hamdy

**Affiliations:** 1 Gulf Medical University

This review has been migrated. The reviewer awarded 4 stars out of 5 This is an excellent paper writing a theoretical, conceptional frame work for developing a Team Work Model for studying Interprofessional Healthcare Teams. The model described has many applications not limited to patient safety alone. It can be used in developing and investigating effectiveness of Interprofessional Healthcare Teams in educations and other fields. The literature is up to date and could be of great value for any scholar who may wants to use the mode in research.I recommend this article as an important contribution to the field of research Teamwork.

Reviewer Expertise:

NA

No decision status is available

# Reviewer report: Reviewer response for version 1

**DOI:** 10.21956/mep.19807.r29808

**Published:** 2019-04-12

**Authors:** Ken Masters

**Affiliations:** 1 Sultan Qaboos University

This review has been migrated. The reviewer awarded 2 stars out of 5 Overall, a mostly well-written paper in which the authors have identified the serious problem of the number of preventable deaths in Healthcare. The authors are also be commended on their detailed discussions of the background issues, such as the definitions and concept of a team, and the development of a Teamwork Model built upon existing theoretical frameworks and insights.There is, however, a serious error in the use and description of Engeström’s model (Figure 3): the error around the use of “Object”, and the lack of an “Outcome”.In their diagram, the “Object” is presented in the sense of “Objective”. For that reason, the authors have overlaid the “Goal: Identification of the patient” on the “Object”, and have conflated Object/Objective/Goal. This mis-alignment of Object and Objective/Goal is then emphasised in the later discussion (e.g. where they speak of “achieve the goal or objectives”).In Engeström’s writings, however, including in the source cited by the authors (Engestrom, Y. (2000) 'Activity theory as a framework for analyzing and redesigning work', Ergonomics, 43(7), pp. 960-974. https://doi.org/10.1080/001401300409143), Object is not used in this sense. It is used more in the grammatical sense of Subject-Verb-Object, where the Subject is the person performing the action, and the Object is the person being acted upon. In Engeström’s work, there is an extension via an arrow to an “Outcome”, which is a goal. For example, in his work, Engeström has the following: • Subject: Physician; Object: Patient Records; Outcome: Image of the task.• Subject: Physician; Object: Patient and his father; Outcome: Preliminary Assessment.• Subject: Physician; Object: Lung Specialist; Outcome: Promise to come.• Subject: Lung Specialist; Object: Patient and his father; Outcome: Decision to admit the patient.So, in their model, where the authors have:• Subject: OR Nurse; Object: GOAL: Identification of the Patient.They should rather have:• Subject: OR Nurse; Object: Patient[?]; Outcome: Identification of the Patient.While this is a useful paper, the authors are setting themselves up for severe criticism if they leave these Figures as they are. I strongly recommend that, after they have had a chance to see other reviewers’ comments, they take the opportunity to publish a Version 2 of the paper in which they:• Recreate Figure 2 and Figure 3 to more faithfully reflect Engeström’s work;• Adapt their terminology to avoid confusion (e.g. use “Object” in the sense that Engeström uses it, and, instead of “Objective” or “Goal”, use Engeström’s “Outcome”), and • Carefully go through their model again, ensuring that their final product aligns with Engeström’s work.

Reviewer Expertise:

NA

No decision status is available
